# De novo expression of gastrokines in pancreatic precursor lesions impede the development of pancreatic cancer

**DOI:** 10.1038/s41388-022-02182-4

**Published:** 2022-01-26

**Authors:** Sabrina Steiner, Gitta M. Seleznik, Theresia Reding, Matea Stopic, Daniela Lenggenhager, Emiel ten Buren, Dilmurodjon Eshmuminov, Katharina Endhardt, Catherine Hagedorn, Anna M. Heidenblut, Anna Bratus-Neuenschwander, Jonas Grossmann, Christian Trachsel, Karolina S. Jabbar, Stephan A. Hahn, Johannes vom Berg, Rolf Graf, Anurag Gupta

**Affiliations:** 1 Visceral & Transplantation Surgery, University Hospital Zürich, 8091 Zürich, Switzerland; 2 Department of Pathology and Molecular Pathology, University Hospital Zürich and University of Zürich, 8091 Zürich, Switzerland; 3 Institute of Laboratory Animal Science, University of Zurich, 8952 Schlieren, Switzerland; 4 Faculty of Medicine, Department of Molecular GI Oncology, Ruhr University of Bochum, 44780 Bochum, Germany; 5 Functional Genomics Center Zurich, University of Zurich, ETH, 8093 Zurich, Switzerland; 6 Department of Medical Biochemistry, University of Gothenburg, 405 30 Gothenburg, Sweden

**Keywords:** Cancer models, Pancreatic cancer

## Abstract

Molecular events occurring in stepwise progression from pre-malignant lesions (pancreatic intraepithelial neoplasia; PanIN) to the development of pancreatic ductal adenocarcinoma (PDAC) are poorly understood. Thus, characterization of early PanIN lesions may reveal markers that can help in diagnosing PDAC at an early stage and allow understanding the pathology of the disease. We performed the molecular and histological assessment of patient-derived PanINs, tumor tissues and pancreas from mouse models with PDAC (KC mice that harbor K-RAS mutation in pancreatic tissue), where we noted marked upregulation of gastrokine (GKN) proteins. To further understand the role of gastrokine proteins in PDAC development, GKN-deficient KC mice were developed by intercrossing gastrokine-deficient mice with KC mice. Panc-02 (pancreatic cancer cells of mouse origin) were genetically modified to express GKN1 for further in vitro and in vivo analysis. Our results show that gastrokine proteins were absent in healthy pancreas and invasive cancer, while its expression was prominent in low-grade PanINs. We could detect these proteins in pancreatic juice and serum of KC mice. Furthermore, accelerated PanIN and tumor development were noted in gastrokine deficient KC mice. Loss of gastrokine 1 protein delayed apoptosis during carcinogenesis leading to the development of desmoplastic stroma while loss of gastrokine 2 increased the proliferation rate in precursor lesions. In summary, we identified gastrokine proteins in early pancreatic precursor lesions, where gastrokine proteins delay pancreatic carcinogenesis.

## Introduction

Patients with pancreatic ductal adenocarcinoma (PDAC) show a poor overall 5-year survival rate of less than 5% after the diagnosis [1]. Approximately 80% of PDACs are detected at advanced metastatic stages [1], when current multimodal cancer treatments remain ineffective [2]. In PDAC pathology, pancreatic intraepithelial neoplasia (PanIN), intraductal papillary mucinous neoplasm (IPMN), and mucinous cystic neoplasm (MCN) are considered pre-malignant precursor lesions [3]. PanINs, the most frequent pre-malignant lesions, are classified into low-grade and high-grade PanINs. Structurally, low-grade PanINs have a flat or papillary mucinous epithelium without high-grade cellular atypia while high-grade PanINs correspond to carcinoma in situ [3]. Studies suggest a progression model for PanIN development, where the consecutive accumulation of genetic changes parallels the histological grade of neoplasia [4, 5]. Therefore, molecular analysis of PanIN development may reveal markers that could facilitate the understanding of PDAC pathology.

The most common oncogenic mutations associated with all PDAC stages are found in the *KRAS* gene; however, signaling pathways driving the progression of precursor and invasive lesions are not yet fully understood [6, 7]. The *KRAS* gene driven KC (*p48* + */*^*Cre*^*; Kras* + */*^*G12D*^) mouse model, a well-established model for pancreatic carcinogenesis, recapitulates the morphological features of PDAC development [5]. Studies using the KC mice also postulate a stepwise progression of PDAC, where first acinar cells undergo ductal reprogramming- known as acinar-to-ductal metaplasia (ADM)—followed by PanIN lesions leading to invasive pancreatic cancer [8].

Gastrokines (GKNs) are predominantly stomach-derived secretory proteins that maintain homeostasis of the gastric mucosa. GKNs have also been reported in the placenta and duodenum [9, 10]. Gastrokines are comprised of three paralogs: GKN1, GKN2, and GKN3. Among those, GKN1 is known to encode an 18 kDa antral mucosal protein, which is thought to act as a gastric cell-specific cytokine [11]. GKN2 also encodes a functional protein while in humans GKN3 exists as an inactive pseudogene, whereas its homolog is detectable in mice [9]. The absence of GKNs in gastric cancer tissues led to the perception that these proteins act as tumor-suppressors [9]. A recent study has shown that loss of GKN2 can promote gastric inflammation and tumor progression [12].

In this study, while screening for molecular changes occurring during PanIN to PDAC development in transgenic KC mice, we noticed the progressive expression of gastrokine 1 and 2 within the pancreas of mice. Since gastrokine proteins were not described before in PDAC pathology, we explored the role of these proteins from the early to late stages of PDAC development. Upon further characterization, we noticed the expression of gastrokine 1 and 2 in mouse and human pre-malignant early PanIN lesions, secretion of these proteins in pancreatic juice and in serum of KC mice. Furthermore, loss of gastrokine proteins accelerated the development of PanIN lesions and increased cancer incidences. Mechanistically, we noted that GKN1 and GKN2 prevent pancreatic cancer development via different modes of action. Loss of GKN1 led to earlier carcinogenesis by delaying apoptosis and additionally inducing desmoplastic stromal changes, while loss of GKN2 increased the proliferation rate in PanIN lesions.

## Results

### Detection and localization of gastrokines in mouse and human pancreas

**Fig. 1:**
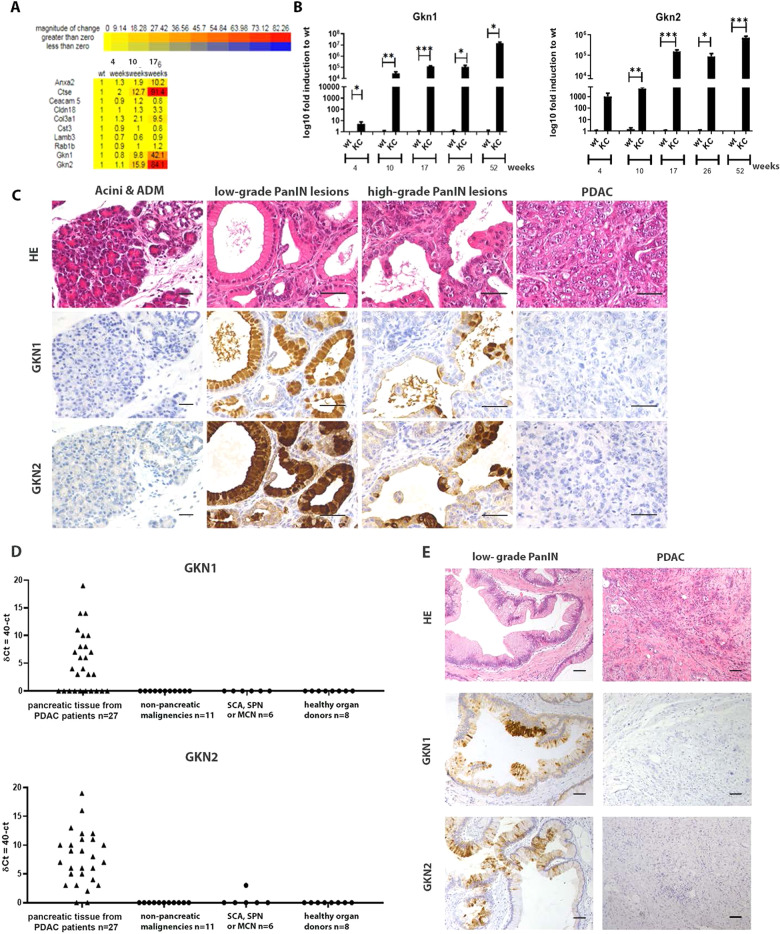
Gastrokine expression in pre-malignant lesions of KC mice and in human pancreas samples. **A** Selected genes from the microarray analysis of KC mice (at 4, 10, and 17 weeks of age). **B** Temporal analysis of *Gkn1* and *Gkn2* transcripts in KC mice by qRT-PCR (one-way ANOVA). **C** H&E, GKN1, and GKN2 staining in KC mice (scale bars 50 µm). Acini and ADM visualized in 10 weeks old; low- and high-grade PanINs in 9 months old and PDAC in 12 months old KC mice. **D** *GKN1* and *GKN2* mRNA transcripts from patients undergoing pancreatic surgery due to lesions in the pancreas compared to healthy control pancreas. Expression levels are reported as dCT (40-Ct). (χ²-test malignancy: *GKN1* 12.53 > 3.84, *GKN2* 29.77 > 3.84; adenoma: *GKN1* 7.79 > 3.84, *GKN2* 10.92 > 3.85) **E** H&E, GKN1, and GKN2 immunohistochemistry in human low-grade PanINs and PDAC (scale bar 100 μm).

To examine gene transcripts in stepwise PanIN to PDAC development, we performed a whole genome microarray analysis of the pancreas of *p48* + */*^*Cre*^*; Kras* + */*^*G12D*^ (KC) mice (https://www.ncbi.nlm.nih.gov/geo/ ID; GSE164620). In the pancreas of 4, 10, or 17 weeks old KC mice, we observed prominent continuous upregulation of *Gkn1* and *Gkn2* (Fig. 1A), paralleling to the age of the mice. Given that we detected gastrokines in the pancreas of KC mice, we further characterized gastrokine expression in the pancreas of KC mice and in human pancreatic biopsies.

We assessed the pancreas from KC mice at different time points (4, 10, 17, 26, and 52 weeks) using quantitative (qPCR) and qualitative (IHC) methods. Similar to the microarray data, qPCR analysis showed marked upregulation of gastrokines in the mouse pancreas as early as 4 weeks, gradually increasing over time (Fig. 1B). This increase paralleled with the progression of pre-malignant lesions in KC mice (Supplementary Fig. 1A). IHC analysis showed co-expression of GKN1 and GKN2 in PanIN lesions, with strong positivity in the cytoplasm of dysplastic epithelium (Fig. 1C, Supplementary Fig. 1B). Further, we could confirm a correlation of GKNs expression with corresponding transcription factor NKX6.3 in PanIN lesions of KC mice (Supplementary Fig. 1C). Selective assessment of low- and high-grade PanINs elucidated strong and diffuse expression of gastrokines in low-grade PanINs, whereas only focal expression in high-grade PanINs (Fig. 1C). Interestingly, GKNs were not detectable in normal pancreas, ADM, and invasive carcinoma (Fig. 1C).

Clinical value for gastrokine proteins within pancreatic lesions of mice seemed relevant when we reevaluated an old data set from a comprehensive gene expression repository of human PanIN lesions via aRNA-longSAGE analysis. In this analysis, individual single-cell pools from microdissected PanIN-1B, PanIN-2, and PanIN-3 cells were compared with pooled normal pancreatic ductal cells. A Venn diagram (Supplementary Fig. 2A) illustrates the overlaps of differentially expressed genes among the PanIN grades. In this analysis, GKN1 expression showed prominent specificity in early PanIN lesions (62% and 17% of PanIN-1B and −2 lesions, respectively) as it was not observed in acinar cells, PanIN-3 cells, or in PDAC cells (Supplementary Fig. 2B). *GKN1* expression was also confirmed by qRT-PCR on microdissected samples from individual patients containing cells of normal acini, PanINs, and PDAC (Supplementary Fig. 2C). In this data set, *GKN2* was not detected due to the limited number of expression tags available at that time.

To validate further aRNA-longSAGE data and to detect *GKN2*, we characterized and quantified gastrokines in an independent cohort of patient samples. We analyzed pancreatic tissues from patients, who underwent pancreatic surgery at the University Hospital Zurich (Supplementary Table 1). qPCR analysis showed upregulation of gastrokine transcripts in tumor tissue samples from PDAC patients. Of note, the site of tissue sampling was the tumor itself and peri-tumoral tissue (adjacent or distant from tumor) (Supplementary Table 1) (Fig. 1D). Interestingly, no *GKN* expression was observed in and around samples of other pancreatic lesions (e.g., SCA, SPN, or MCN), in lesions of non-pancreatic origin (e.g., metastasis), and in healthy pancreas tissue. In contrast, in and around PDAC samples, there was a frequent co-expression of *GKN1* and *GKN2*. Overall, 52% of studied PDAC samples were positive for *GKN* expression; among them, 41% co-expressed *GKN1* and *GKN2*, whereas 12% only showed *GKN2* expression. Similar to mouse pancreatic tumors, immunohistology analysis revealed GKN expression only in early PanIN lesions of human tumor samples (Fig. 1E).

**Fig. 2:**
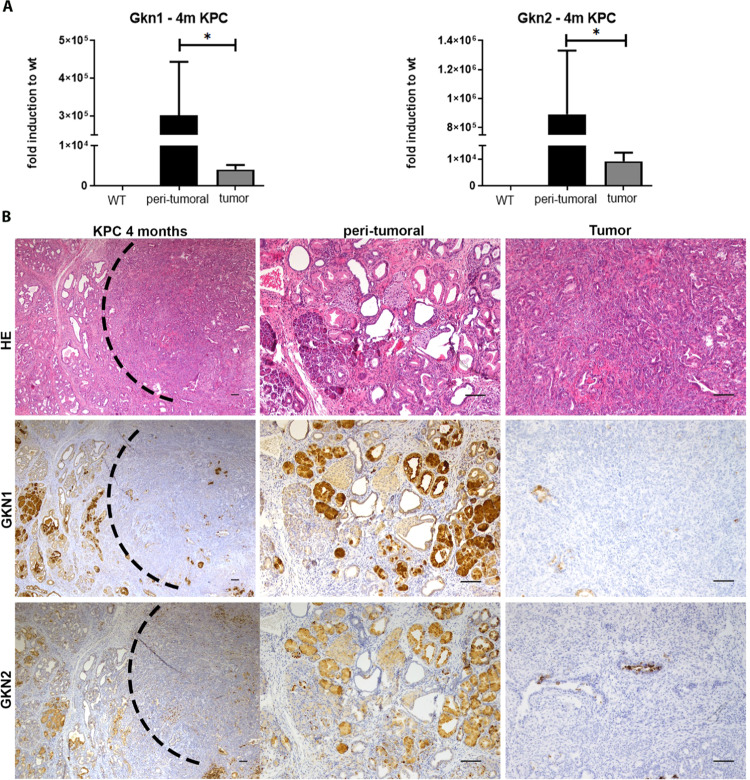
Gastrokine expression in KPC mice. **A** *Gkn1* and *Gkn2* transcripts in 4 months old KPC mice compared to age matched wild-type mice. **B** Visualization of tumor and peri-tumoral tissue in 4 months old KPC mice. H&E, GKN1, and GKN2 immunohistochemistry in consecutive cuts (scale bars 100 µm).

The KC mouse model mimics the development of precursor malignant lesions in the pancreas. However, only 10–15% of KC mice develop invasive pancreatic cancer at later age (between 9–15 months) and high-grade PanIN lesions are relatively rare. To test the presence of GKNs in mice PDACs, we analyzed tumor samples from KC mice lacking p53 tumor suppressor (referred as KPC mice: *p48*^*+/Cre*^*; Kras*^*+/G12D*^, *p53*^*flox/+*^). In this model, tumor development starts at 3 months of age and almost 100% KC mice develop invasive pancreatic cancer [6]. In the KPC model, we evaluated GKN expression within PDAC and in peri-tumoral tissue, which contains a high amount of PanIN lesions. The qPCR analysis showed abundant expression of *Gkn1* and *Gkn2* in peri-tumoral tissue compared to negligible expression in the PDAC tissue (Fig. 2A). Immunohistochemical analysis showed that *Gkn* expression was predominantly associated with low-grade PanIN lesions and no expression in PDAC and high-grade PanIN lesions. These observations corroborate with the findings from the KC model and human aRNA-longSAGE analysis (Fig. 2B). Additionally, western blot analysis also confirmed the presence of GKN1 and GKN2 proteins in the peri-tumoral samples (Supplementary Fig. 3A).

### Gastrokines expression is inflammation independent

**Fig. 3:**
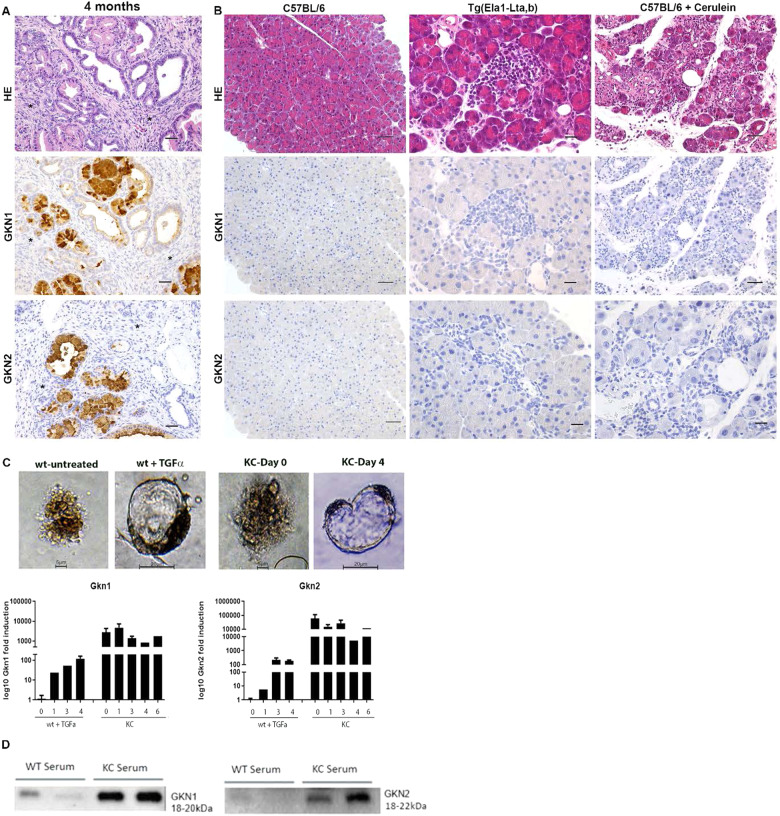
Gastrokine expression during pancreatic inflammation, in vitro in acinar cells and its detection in pancreatic juice. **A** Infiltrating inflammatory cells (*) around the PanIN lesions in KC mice. Immunohistochemical GKN1 and GKN2 positivity, while the inflammatory cells are negative (scale bars 50 µm). **B** H&E, GKN1, and GKN2 staining in C57BL/6 mice (scale bar 100 µm) and in different models of pancreatic inflammation (scale bars 50 µm). The histological images of cerulein treatment represents the chronic treatment regimen. **C** Light microscopy photos of isolated acinar cells embedded in collagen matrix from wt and KC mice at day 0 and day 4. *Gkn1* and *Gkn2* gene expression of KC acinar cells compared to untreated wild type acinar cells. **D** Gkn1 and Gkn2 protein expression on western blot in serum of 9 months old wild type and KC mice.

PanINs are generally accompanied by a prominent inflammatory reaction in the mouse pancreas [13]. Therefore, we assessed whether inflammation is driving *Gkn* expression within PanINs. We noticed that infiltrating inflammatory cells in PanINs do not express GKNs as shown by IHC (Fig. 3A). Furthermore, inflammation is known to promote the development of pre-malignant lesions and PDAC [14]. Therefore, we analyzed *Gkn* expression in various established mouse models of acute and chronic pancreatitis by qPCR (data not shown) and IHC. The analysis of pancreatic inflammation models such as cerulein (CCK analog)-mediated pancreatitis [15] or induction of pancreatic inflammation upon lymphotoxin overexpression (*Tg(Ela1-Lta,b) mice)* [16] revealed no GKN expression (Fig. 3B).

### Gastrokines increase during ductal transdifferentiation

Using a 3D mouse primary acinar cell culture model, we induced acinar-to-ductal cell transdifferentiation in vitro by treating acinar cells with the EGFR ligand TGF-α. In this assay, acinar cells rapidly undergo cyst formation, where acinar genes are silenced and ductal genes begin to express, which are characteristic for the generation of in vivo ADM (acinar-to-ductal metaplasia)/PanIN lesions [17]. In this assay, we noticed that primary acinar cells obtained from wild-type mice do not express Gkn1 and Gkn2. However, during in vitro transdifferentiation, expression of gastrokine transcripts appear progressively in parallel to the number of cysts in the culture (Fig. 3C), supporting the role of gastrokines in acinar transdifferentiation. Interestingly, primary acinar cells isolated from 4 weeks old KC mice steadily expressed *Gkn1* and *Gkn2*, implying that gastrokines expression renders acinar cells susceptible to transdifferentiation.

### Detection of gastrokines in the pancreatic juice and serum

In healthy stomach, GKNs are secreted into the gastric juice. In the pancreas, we found that GKNs are expressed in PanIN lesions that are connected to the pancreatic ductal system. Therefore, we examined the presence of gastrokines into the pancreatic juice of KC mice. We collected pancreatic juice samples from wild-type and KC mice. The presence of GKNs into the pancreatic juice was evaluated with mass spectrometry (MS). MS analysis confirmed the presence of GKN1 and GKN2 proteins in the pancreatic juice of KC mice but not in wild-type mice (Supplementary Fig. 3B). Although gastrokines are likely to be concentrated in pancreatic juice, such samples are difficult to procure in clinical settings involving an invasive procedure. Therefore, serum can be used in clinical settings to assess gastrokines concentrations. We could detect increased concentrations of GKN1 and GKN2 in serum of KC mice using western blotting (Fig. 3D) suggesting that serum can be used to determine gastrokines concentrations in patients samples.

### Loss of gastrokines accelerates PanIN and pancreatic cancer development

**Fig. 4:**
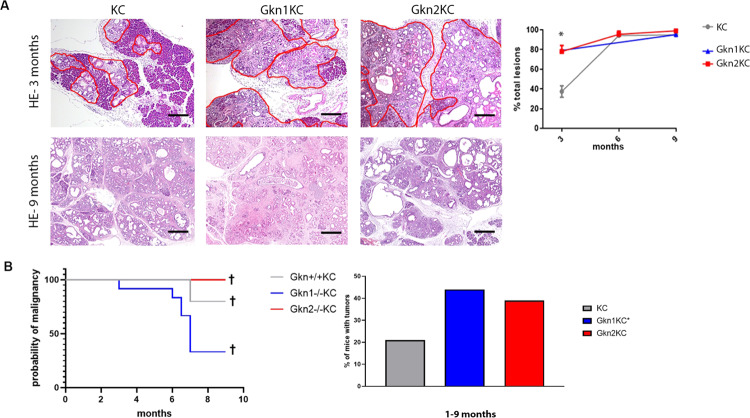
Early lesions and tumor incidence. **A** Representative H&E stainings showing 3 and 9 month old pancreata from KC, Gkn1KC and Gkn2KC mice. Lesion occupied area is framed in red (scale bars 200 µm). Right panel shows area quantification of occupying lesions at 3, 6, and 9 months time points (one-way ANOVA). **B** Time dependent probability of malignant occurrence curve displays all animals presenting malignancy. Mice were sacrificed when termination criteria were met or 9 month’s time point was reached; (Gehan–Breslow–Wilcoxon analysis). Right panel shows % of mice harboring a tumor KC = 21%, Gkn1KC = 44% and Gkn2 = 39%. * including Gkn1 heterozygous-KC.

To understand the role of gastrokine proteins in PanIN and pancreatic cancer development, we intercrossed gastrokine deficient (*Gkn1*^*−/−*^ and *Gkn2*^*−/−*^*)* mice with KC mice (breeding scheme illustrated in Supplementary Fig. 4). Herein and after, *Gkn1*-deficient KC mice are referred to as Gkn1KC and *Gkn2*-deficient KC mice as Gkn2KC, respectively. We examined the pancreas of KC, Gkn1KC and Gkn2KC mice at 3, 6, and 9 months of age; particularly, we followed PanIN and tumor development. Already at the age of 3 months, almost 80% of the pancreas of Gkn1KC and Gkn2KC mice contained PanIN lesions compared to roughly 40% PanIN lesions in the pancreas of KC mice (Fig. 4A). However, at 6 and 9 months’ time points the PanIN areas within the pancreas of KC, Gkn1KC, and Gkn2KC mice were comparable. This data suggests that the loss of gastrokine proteins led to earlier occurrence of PanIN lesions. The amount of low and high-grade PanIN lesions in all groups at 9 months’ time point remained similar (data not shown). Furthermore, we assessed the incidence of pancreatic cancer, when mice were 9 months old. Our histopathological evaluation of pancreatic tissues revealed two-fold- increase in pancreatic cancer in Gkn1KC and Gkn2KC mice (Fig. 4B). These results suggest that gastrokine proteins prevent tumor development probably by delaying early PanIN progression.

### GKN1 and GKN2 delay PanIN development via apoptosis promotion and reduced cell proliferation, respectively

**Fig. 5:**
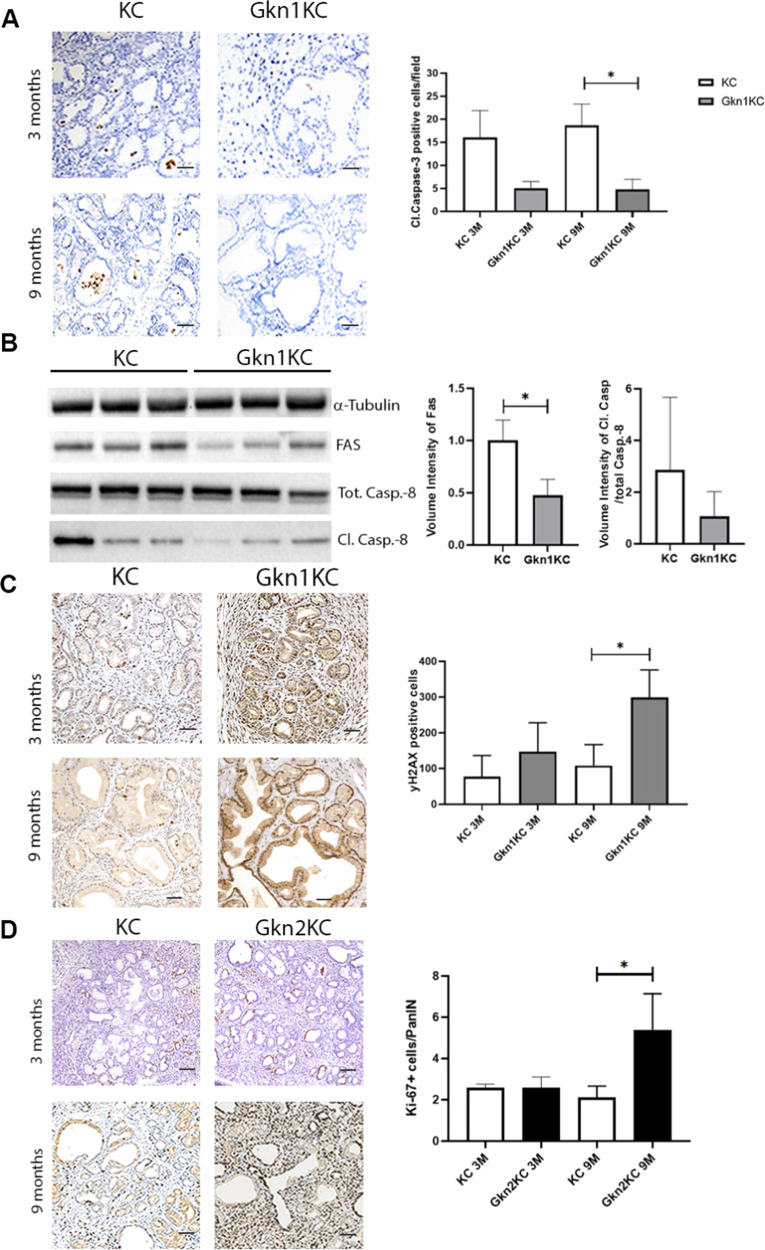
Apoptosis, senescence and proliferation related changes in Gkn1KC and Gkn2KC mice. **A** Cleaved caspase-3 quantification displayed as positive cells/image field for KC and Gkn1KC at 3 and 9 months of age with representative IHC images. **B** Western blot at 3 months, indicating differential regulation of extrinsic apoptotic pathway in Gkn1KC compared to KC pancreatic tissue. Densitometric analysis of FAS (left graph) and Cleaved Caspase-8/ Total Caspase-8. **C** γH2AX quantification shown as positive cells/ image field for KC and Gkn1KC mice at 3 and 9 months of age with representative IHC images. **D** Amount of Ki-67 positive cells per PanIN in Gkn2KC mice at 3 and 9 months (scale bars 100 µm) (one-way ANOVA).

To discern mechanisms behind accelerated PanIN formation and increased PDAC incidence upon gastrokine loss, we performed a qPCR-based screening of pancreatic tissue from KC, Gkn1KC and Gkn2KC mice at 3 and 9 months of age. We analyzed several markers related to PanIN lesions, stromal and tissue remodeling, cell cycle status, proliferation and apoptosis, epithelial to mesenchymal transformation, inflammation and immune cells accumulation (Supplementary Table 2). Compared to KC mice, most of above-mentioned markers were upregulated in the pancreas of Gkn1KC and Gkn2KC; however, the increase was not significant when normalized to the amount of PanIN lesions (not shown). Nevertheless, we found differences in apoptosis markers between KC and Gkn1KC, while Gkn2KC appeared to have an altered cell proliferation response. Compared to KC mice, we noticed fewer cleaved caspase-3 positive cells within PanIN lesions of Gkn1KC mice at 3 and 9 months’ time points suggesting reduced apoptosis (Fig. 5A). A deeper analysis of the apoptosis pathway revealed that GKN1 significantly downregulated FAS and cleaved caspase 8 proteins (Fig. 5B), both members of the extrinsic apoptosis pathways. However, molecules associated with intrinsic apoptosis pathways remained unchanged (Supplementary Fig. 3C). Additionally, 9-months old Gkn1KC mice also showed a significant increase in γH2AX (a marker of cellular senescence and DNA damage) positive cells (Fig. 5C) [18]. Interestingly, compared to KC mice, 9-months old Gkn2KC mice displayed significantly higher number Ki-67^+^ proliferating cells within PanIN lesions (Fig. 5D).

### Stromal changes within the pancreas upon loss of gastrokine proteins

**Fig. 6:**
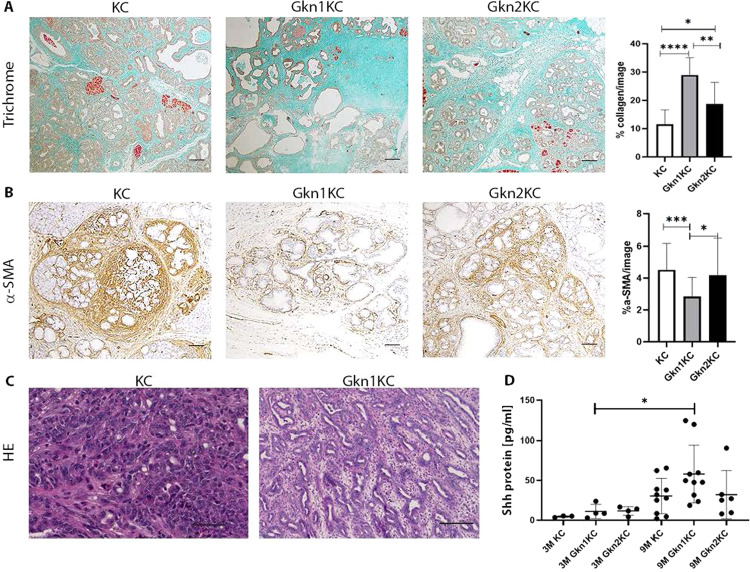
Stromal changes in pancreatic tissue and tumor differentiation status. **A** Masson Trichrome staining on 9 months old pancreata from KC, Gkn1KC, and Gkn2KC mice (scale bars 200 µm; one-way ANOVA). **B** α-SMA positive stained myofibroblast in Gkn1KC, KC, and Gkn2KC tissue at 9 months (scale bars 200 µm; one-way ANOVA). **C** Representative images of KC tumors with solid-glandular growth pattern, and Gkn1KC tumors with better differentiated tubulo-glandular growth pattern (scale pars 100 µm). **D** ELISA of pancreatic tissue homogenate from age matched KC, Gkn1KC and Gkn2KC mice.

The activation of pancreatic stellate cells (PSC) for e.g., via sonic hedgehog (SHH) enhances the accumulation of dense collagen rich stroma during PDAC development [19]. Such stromal changes are thought to be associated with aggressive tumor phenotype and induce chemo-resistance [20–22]. Our histopathological analysis of pancreas tissue with pre-malignant lesions from 9-months old Gkn1KC, Gkn2KC, and KC mice showed an increase in collagen occupied area with reduced number of α-SMA + cells mostly in Gkn1KC mice (Fig. 6A, B), implying that this dense collagen rich environment does not favor myofibroblast growth and maintenance. Further histopathological assessment revealed less differentiated tumors with solid or small-glandular growth pattern in KC mice while Gkn1KC mice showed better differentiated tumors with tubulo-glandular growth pattern (Fig. 6C). This might be influenced by increased concentrations of sonic hedgehog (SHH) protein found in pancreatic tissue of Gkn1KC mice (Fig. 6D) [19].

### GKN1 acts as tumor suppressor in vitro and in vivo

**Fig. 7:**
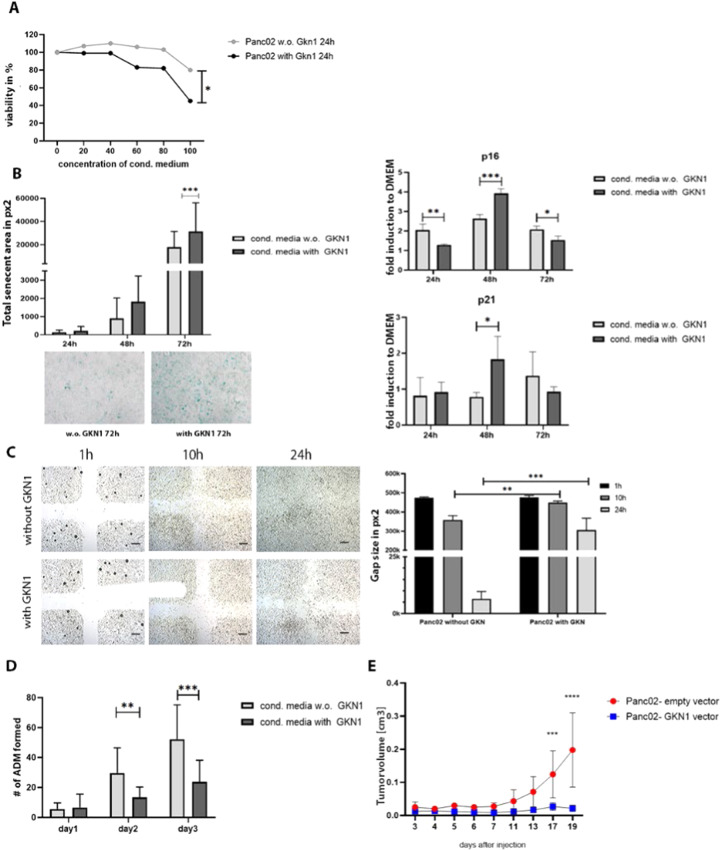
In vitro and in vivo effects of GKN1 protein. **A** Viability of Panc-02 cells was assessed by MTT colorimetric assay after 24 h of incubation with conditioned media containing GKN1 protein. **B** β-Galactosidase staining on Panc-02 cells after incubation with GKN1 or control conditioned medium. Representative pictures of compared groups are shown for the 72 h time point. Expression profile of senescence genes such as p16 and p21 was performed via qPCR analysis on Panc-02 cells treated with conditioned media with or without GKN1. Gene expression was analyzed over 24, 48, and 72 h. **C** Migration assay of Panc-02 cells after 24 h of incubation with GKN1 or control conditioned medium. Representative pictures at 5x magnification show migration over 1, 10, and 24 h time period (scale bars 200 μm). **D** In vitro transdifferentiation of isolated acinar cells from KC mice, treated with conditioned media containing GKN1. **E** Subcutaneous tumor development over 19 days after injection of either Panc-02-empty vector or Gastrokine1^+^ Panc-02 cells.

To further investigate effects of gastrokine proteins on tumor growth, we generated GKN1 overexpressing Panc-02 cells. Gastrokine1^+^ Panc-02 cells showed similar cell proliferation profile as parental Panc-02 cell and secreted GKN1 in the culture medium (not shown). Addition of GKN1 containing conditioned medium (CM) and CM without GKN1 from cultures of Gastrokine1^+^ Panc-02 and Panc-02 cells, respectively, reduced viability of Panc-02 cells in vitro (Fig. 7A) and increased apoptosis of Panc02 cells (Supplementary Fig. 5). The role of GKN1 in senescence via p16 and p21 upregulation has already been shown in the context of gastric cancer [23]. Therefore, we examined p16 and p21 mRNA transcripts in Panc-02 pancreatic cancer cells, treated with conditioned media with and without GKN1. Our results revealed a significant upregulation of p16 and p21, which is likely responsible for growth inhibition/senescence state. We also examined senescence induction in Panc-02 cells by analyzing the expression of β-galactosidase, an established protein marker of cellular senescence. Our results showed a progressive increase over 24–72 h. (Fig. 7B). Furthermore, GKN1 containing CM reduced migration of Panc-02 cells in a time-dependent manner when compared to Panc-02 cells exposed to control CM without GKN1 (Fig. 7C). Our findings related to reduced viability and migration of Panc-02 cells after exposure to GKN1 are in line with published results [24–26]. We further assessed the direct impact of GKN1 containing CM on murine acinar cells and thereby treated primary acinar cells isolated from KC mice with fresh medium without GKN1, or with 50% or 100% GKN1 containing CM. In this assay, we noticed dose-dependent reduction in transdifferentiated acinar structures (Fig. 7D).

Lastly, we subcutaneously injected GKN1^+^ Panc-02 and control Panc-02 cells that were transfected with empty vector in C57BL/6 mice. In comparison to Panc-02 cells with empty vector, GKN1^+^ Panc-02 cells developed significantly smaller tumors (Fig. 7E). Overall, these observations support the notion that GKN1 inhibits the transition from low-grade PanIN to high-grade PanINs; thus, acting as tumor suppressor.

## Discussion

In this study, we report the exclusive association of gastrokine proteins with pre-malignant PanIN lesions of human and mouse pancreas as these proteins were not detected in healthy pancreas or in pancreatic cancer. To unravel the role of these proteins in PanIN lesions we analyzed pancreas of KC mice. The KC mouse model is ideal to study the whole spectrum of development from normal tissue to pre-malignant lesions to invasive pancreatic cancer. A long-term molecular follow up of KC pancreas from one until twelve months of age revealed progressive increase in gastrokine transcripts that paralleled with increased amounts of PanIN lesions. Importantly, we observed a similar expression pattern in the pancreas of PDAC patients, i.e., abundance of these proteins in low-grade PanINs and their absence in normal pancreas tissue, high-grade PanINs and in pancreatic cancer. Therefore, high expression level of gastrokines in low-grade PanINs might reflect a rescue mechanism to maintain the integrity of the pancreatic ductal epithelium perhaps as part of the gastrointestinal repair mechanism. The expression of several gastrointestinal markers in pancreatic lesions has been shown in previous studies [27, 28], but the role of gastrokines in PDAC development was not explored.

The loss of gastrokine proteins in high-grade PanINs and in pancreatic cancer suggests that these structures differ largely at the molecular level from low-grade lesions and may not follow the sequential progression to pancreatic cancer. Some recent opinions debate the stepwise PanIN progression model, as it has been suggested that high-grade PanINs are mostly present adjacent to the invasive tumor and they share cytomorphology with invasive tumor cells that re-populate the normal ductal epithelium, which is called cancerization of ducts (COD) [29]. In some cases, high-grade PanINs can have higher somatic mutational burden than their associated PDACs [30], which supports the hypothesis that a substantial proportion of reported high-grade PanINs in reality might represent CODs; however, histologically, it is not possible to differ between high-grade PanINs and CODs [31].

We detected secreted gastrokine proteins in pancreatic juice and in serum of mice, which may warrant using these proteins as early biomarkers. In a recent study [32], pancreatic cyst fluid samples obtained by routine endoscopic ultrasound-guided aspiration were analyzed by MS. In an exploratory cohort of 24 patients, GKN1 was identified in the cyst fluid of four patients. Two of those were invasive gastric/pancreatobiliary-type IPMN, one PDAC with a cystic component and one was a serous cystic tumor with PanIN lesions in the surrounding pancreas parenchyma. By contrast, GKN2 was not found in any cyst fluid sample. Thus, GKN1 may potentially serve as early biomarkers to suggest predisposition to PDAC or to distinguish between benign and malignant processes, which may provide clinical benefit to some patients, e.g., as surveillance measure for patients with hereditary pancreatic cancer or chronic pancreatitis.

After the discovery of GKN proteins in human and mouse pancreatic lesions, we aimed at revealing the biological role of these proteins during PDAC development. Data obtained from *Gkn1* and *Gkn2* deficient KC mice points to their essential but distinct mechanisms in pancreatic carcinogenesis. Our data indicated that these proteins may inhibit steps of tumor formation as they delay PanIN progression. We could show that GKN1 promotes the extrinsic apoptosis pathway, however, it is not yet clear whether GKN1 is directly involved in the apoptosis machinery or if it is based on an indirect mechanism, for instance, by the upregulation of sensing receptors. In addition, senescence, another mechanism to prevent malignant transformation, could be influenced by GKN1, as suggested by the increased amount of γH2X positive cells in the pancreas of Gkn1KC mice. It has been previously shown in vitro that gastric cancer cells stably expressing GKN1 or being exposed to GKN1 protein, resulted in cellular retention in G1 cell cycle phase. This consequently led to a senescent state and later to apoptosis [23, 33]. As constitutively activated *Kras* causes oncogene induced senescence in KC mice [34], we speculated that GKN1 is able to bypass the senescent state and force gastrokine positive cells into apoptosis. Of note, mice with stable GKN1 expression (KC or Gkn2KC) had reduced number of senescent cells and increased amount of apoptotic cells, when compared to Gkn1KC mice. Furthermore, loss of GKN1 had an impact on tumor stroma leading to the development of a denser and highly collagen rich stroma. This seems to affect the invasiveness, i.e., histomorphologically, those tumors were better differentiated with mostly tubulo-glandular growth patterns, hinting to a less aggressive phenotype. Our in vitro results support the hypothesis that secreted GKN1 protein acts as tumor suppressor by changing the invasive capacity of cancer cells and preventing transdifferentiation of primary acinar cells. Furthermore, inoculation of GKN1 ^+^ cancer cells in mice significantly hindered tumor progression supporting the role of GKN1 as a tumor suppressor. In contrast, we propose that GKN2 delays PanIN progression via impaired proliferation of PanIN lesions.

What triggers gastrokine expression in the pancreas remains unclear? The particular dynamics of gastrokine expression (no expression in normal pancreatic tissue – high-expression in low-grade precursor lesions –loss of expression in high-grade precursor lesions and invasive cancer) remains to be explored in future studies. Our pancreatic inflammation mouse models seem to rule out the role of inflammatory reactions in gastrokine induction. The effect of inflammation in pre-malignant lesions in humans may differ from our tested mouse models needing further investigations. A further open question is whether ADMs are essential for gastrokine expression. Our in vitro transdifferentiation studies revealed a gradual increase of gastrokines in wild-type acinar cells through acinar-to-ductal transdifferentiation. However, acinar cells from KC mice already expressed gastrokine proteins starting from day 0 with a constant increase over time, suggesting alternative mechanisms.

In summary, the results of this study show that gastrokines are overexpressed in early pancreatic cancer precursor lesions and delay carcinogenesis. Detection of gastrokine proteins in PanIN lesions, in the pancreatic juice and in the serum of mice is inspiring to explore the diagnostic potential of these proteins in patients with PDAC. In which way these proteins could play a role as biomarkers for an earlier detection of PDAC needs further investigation with appropriate number of human samples from different pathological stages.

## Methods

### Mice

Mice were maintained under specific pathogen-free conditions. The Swiss Animal Protection Law and Veterinary office of Canton Zurich approved experiments. C57BL/6 (WT) and B6.129S4-Kras<tm4Tyj > /J(#008179) (KC) and B6.129P2-Trp53 < tm1Brn > /J(#008462) (KPC) mice were purchased from The Jackson Laboratory, B6.129-Ptf1a < tm1(cre)Cvw> from repository of Mutant *Mouse* Resource & Research Centers (NIH, USA). The wild type (+/+) controls and all genotype groups analyzed in the study were littermates and bred on the same mixed C57BL/6 x B6.129S4-Kras<tm4Tyj > /J(#008179) x B6.129P2-Trp53 < tm1Brn > /J(#008462) x B6.129-Ptf1a < tm1(cre)Cvw > /Mmnc background. C57BL/6N-Gkn1 < tm1(KOMP)Vlcg> and C57BL/6N-Gkn2 < tm1(KOMP)Vlcg> complete knockout mice were used for intercrossing with mice B6.129S4-Kras<tm4Tyj > /J(#008179) and B6.129-Ptf1a < tm1(cre)Cvw > /Mmnc to generate Gkn1KC and Gkn2KC mice. Corresponding controls were used in the form of WT, Gkn1-/- and Gkn2-/- mice (without KC) and KC mice were used as positive controls. All genotype groups analyzed were littermates and bred on the same mixed background. Native blood samples for serum analyses were collected by heart puncture. The breeding scheme of mice is illustrated in Supplementary Fig. 4.

### Human pancreas samples

Human pancreatic tissues were collected at the University Hospital Zurich. The Ethics Committees of the University Hospital Zurich and the Canton of Zurich (Ref. Nr.StV 26-2005) authorized the research project. The study protocol was in accordance with the ethical guidelines of the Helsinki declaration. For the SAGE analysis, informed consent was obtained from all patients undergoing surgery and the ethics committee at the University of Kiel approved the trial.

### RNA extraction

RNA was extracted as described previously [14] or with a newly established method using the Precellys®24 Dual homogenizer (Berlin) with MagNA Lyser Green Beads (Roche Applied Science). In short, a small piece of snap frozen tissue was transferred to a tube containing beads, 650 µl of Lysis buffer (Qiagen) was immediately added and the tube immediately transferred to the Precellys and homogenized once at 6000 rpm for 30 s. RNA was extracted following the Qiagen RNeasy Mini Kit extraction protocol with an on column DNase digestion step. Purified RNA was reversely transcribed into cDNA using qScript™ cDNA SuperMix (Quantabio) according to the manufacturer’s protocol.

### Real-time polymerase chain reaction

For mRNA expression analysis, real-time PCR was performed with TaqMan® Gene Expression Assays (Applied Biosystems, AB). Real-time PCR was run on a 7500 Fast Real-Time PCR System) using TaqMan® Fast Universal PCR Master Mix (Applied BiosystemsTM) under standard conditions. Transcript levels were quantified using 18 S RNA (Applied Biosystems) as a reference and normalized.

### Histology and immunohistochemistry

Paraffin (3 μm) sections of pancreas samples were stained with Hematoxylin/Eosin, Masson trichrome, or various primary and secondary antibodies. Paraformaldehyde (4%) fixed and paraffin embedded tissues were stained on an Autostainer Link 48 (Dako, Glostrup, Denmark). Primary antibodies used in this study were: Human/Mouse Gkn1 antibody from bio-techne R&D Systems (AF7287), anti-Gastrokine 2 from Abcam (ab188866), GAPDH from Santa Cruz (sc-25778), α-Tubulin from Cell Signaling (11H10), α-SMA from Cell Signaling (2125), Cleaved Caspase-3 from Cell Signaling (9661 S), Cleaved Caspase-8 from Cell Signaling (8592), total Caspase-8 from Cell Signaling (4927), FAS from Santa Cruz (sc-1024), Ki-67 from Abcam (16667), Bcl-xl from Cell Signaling (2762), Bcl-2 from Cell Signaling (2876), Mcl-1 from Cell Signaling (5453) and γH2AX from Novus Biologicals (NB100-384). Secondary antibodies used in this study were: anti-Rabbit from Dako EnVision+ System-HRP (K4011), rabbit-anti-Sheep Immunoglobulins/HRP from Dako (P0163).

### Three-dimensional (3D) in vitro ADM formation assay

Acinar cells were isolated from 4–5 week-old mice and embedded in collagen matrix as described previously [15]. Acinar to ductal transdifferentiation events in wild type acinar cells were induced by addition of 50 ng/ml recombinant rhTGF−α (R&D Systems). ADM events were counted daily. Cultures were maintained in a 37 °C and 5% CO2 incubator for 6 days with daily medium replacement.

### Generation of GKN1 overexpressing Panc-02 cells

A GKN1 CDS was ordered in a plasmid backbone at integrated DNA technologies, while plasmids pLenti CMV Puro DEST (w118-1) was a gift from Eric Campeau & Paul Kaufman (Addgene plasmid # 17452) [35], pCMV-dR8.2 dvpr was a gift from Bob Weinberg (Addgene plasmid # 8455) and pCMV-VSV-G was a gift from Bob Weinberg (Addgene plasmid # 8454) [36] used for viral transduction. CDS was removed by EcoRV and cloned into pLenti plasmid via gibbson assembly. Lentiviral transfection was carried out in HEK293T cells before using cell medium for transduction of Panc-02 cells.

### In vitro analysis of cells treated with GKN1 protein in conditioned media

The medium from GNK1 transduced Panc-02 cells or Mock transfected Panc-02 cells was mixed with fresh DMEM to generate conditioned medium at various concentrations. The prepared conditioned media were used to treat non transduced Panc-02 cells.

### Migration assay

The assay was conducted by placing a 4-well silicone insert (Ibidi, Gräfelfing, Germany) under sterile conditions into the middle of each well within a 12-well cell culture plate. Cells were seeded at a final concentration of 1000 cells per silicone compartment. After overnight incubation or as soon as cells were confluent, the silicone frame was carefully removed with sterile forceps. Wells were carefully washed with 1 ml PBS before the addition of 2 ml DMEM-GlutaMAX,10% FBS and antibiotic supplement. To analyze migration velocity, a picture of each well was taken for every hour at 5x magnification. Gap size was quantified with NIS-Elements BR Analysis 4.20.02 (Nikon, Japan) imaging software.

### Subcutaneous tumor cell injection

Mice were anesthetized with isoflurane in an enclosed container (5 vol% isoflurane in O_2_). Tumor cells were suspended to a final concentration of 1 × 10^6^/0.1 ml PBS and injected with a 30 G needle into the shaved lower back/flank of 6-week old mice. Tumor size was assed twice a week non-invasively with a caliper and volume was calculated by measurement of tumor length (a) and tumor width (b) and the formula of 4/3* (3.14* a/2 (b/2 ^2)) or in case of sphere shaped tumors 4/3* (3.14* a/2^3). Termination criteria were met when a tumor size of 1.5 × 1.5 cm was reached.

### Statistical analyses and software

GraphpadPrism version 5 (LaJolla, Ca) was used to construct figures and diagrams. One-way ANOVA (results displayed as mean ± standard deviation), unpaired *t*-tests and Gehan–Breslow–Wilcoxon analysis were used where appropriate. Differences were considered statistically significant if *p* < 0.05 and marked with an asterisk. To determine significance of gastrokines in patient samples with PDAC χ²-test was performed. Statistical analysis (*Z*-test) for the longSAGE data was carried out with the program SAGEstat [16] prior to normalization of the tag counts.

## Supplementary information

Supplementary file
