# Supplementary material for: De novo expression of gastrokines in pancreatic precursor lesions impede the development of pancreatic cancer
Source: Oncogene. 2022 Jan 26;41(10):1507–17. doi: 10.1038/s41388-022-02182-4 (PMC8897191; doi:10.1038/s41388-022-02182-4)
Supplement: Supplementary file 1 — Supplementary file [file 41388_2022_2182_MOESM1_ESM.docx]

##
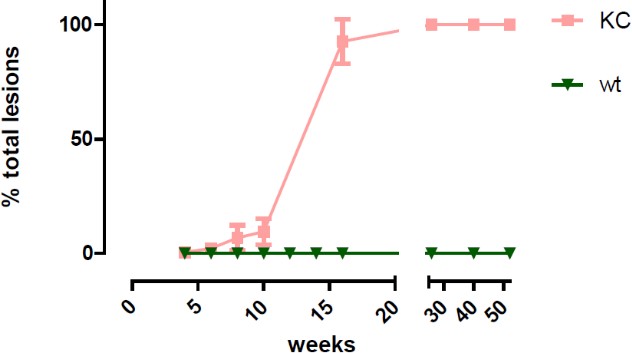
A

**Supplemental Figure 1**

**B**


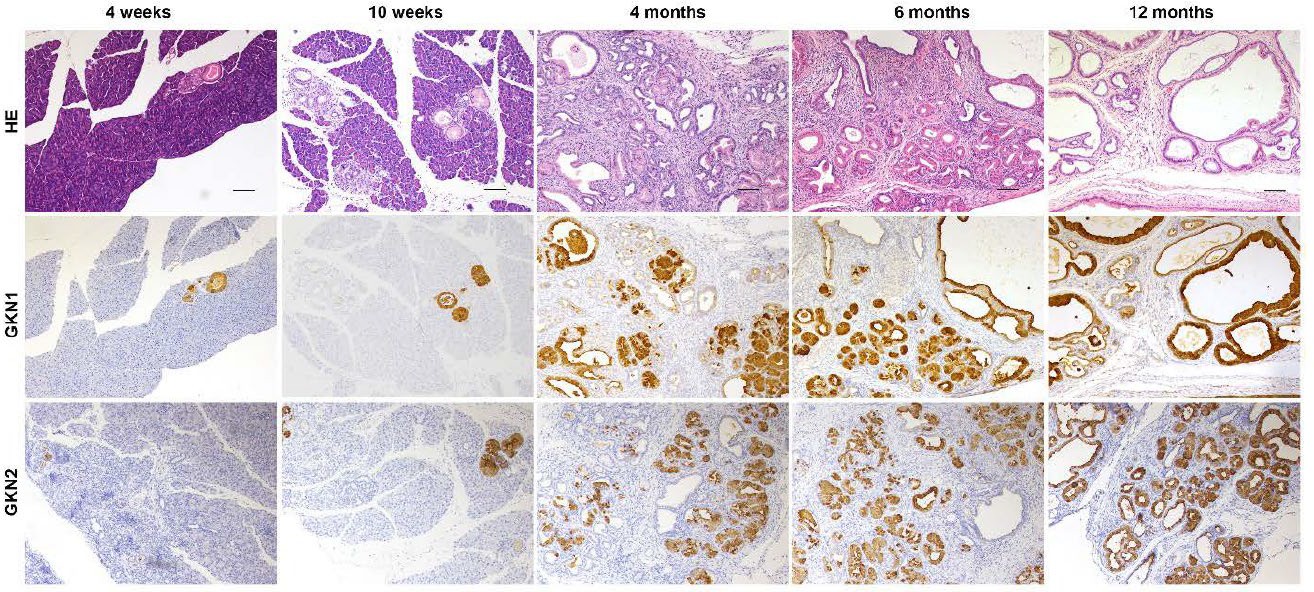


**C**


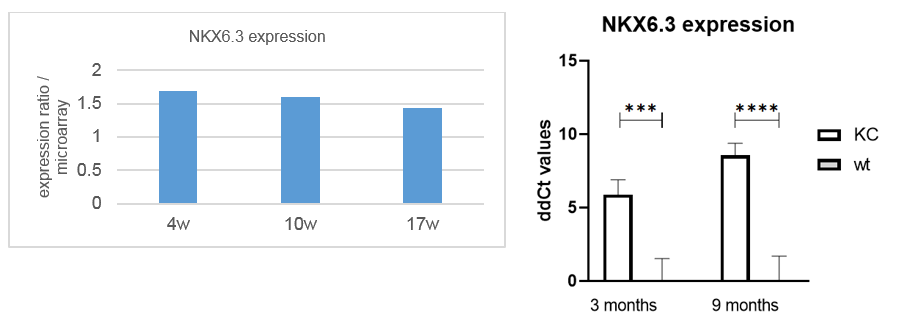


**Supplemental Figure 1**

**Supplemental Figure 1: (A)** Quantification of the percentage of lesions, including ADM and PanIN lesions in KC (n=5) and wild type (n=5) mice over time. 100% represents the complete pancreas. **(B)** The development of premalignant PanIN lesions in KC mice is visualized on HE staining (scale bar 100μm) over time. GKN1 and GKN2 protein expression is shown corresponding to the HE time points. **(C)** NKX6.3 expression ratio in microarray analysis and qPCR based expression in 3 month old KC (n=5) or wt (n=5) and 9 month old KC (n=5) or wt (n=5).

##
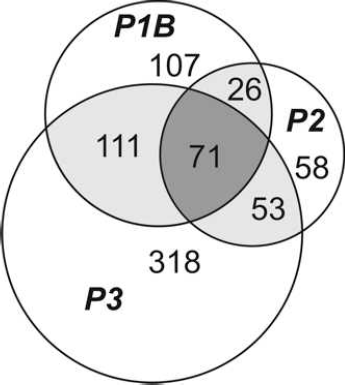

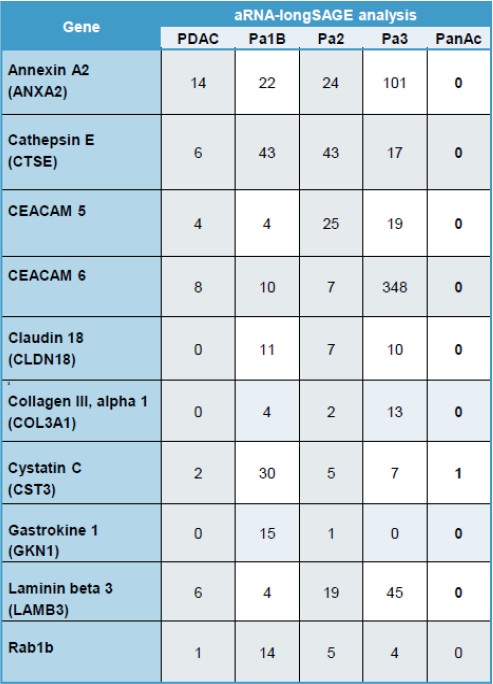
A B

**C**


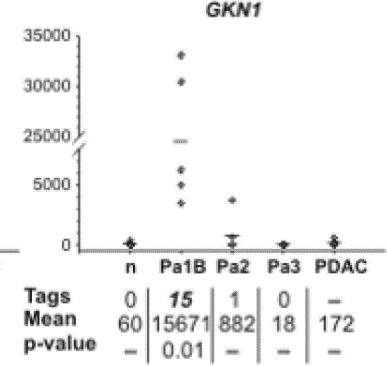
**Supplemental Figure 2**

**Supplemental Figure 2: (A)** Venn diagram of differentially expressed genes in PanINs in comparison to pancreatic normal ductal cells. The overlap between genes differentially expressed with p<0.01 in PanIN-1B (P1B), PanIN-2 (P2) and PanIN-3 (P3) cells is shown. **(B)** Table, with selected genes that were upregulated in the SAGE study of human PanINs. **(C)** Quantitative RT- PCR of GKN1 transcript of the PanIN tissues used in the SAGE analysis. The *y*-axis represents expression levels relative to the sample with the lowest detectable expression of the respective gene. p-values derived from the statistical analysis of the expression differences between sample groups are given only if they reached a threshold of p≤0.05. n, normal pancreatic ductal cells; Pa1B, PanIN-1B; Pa2, PanIN-2; Pa3, PanIN-3; PDAC, pancreatic ductal adenocarcinoma.

## A


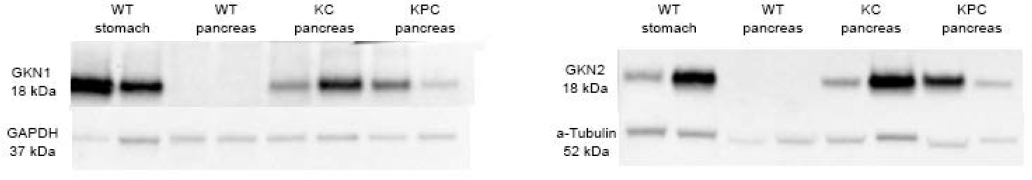


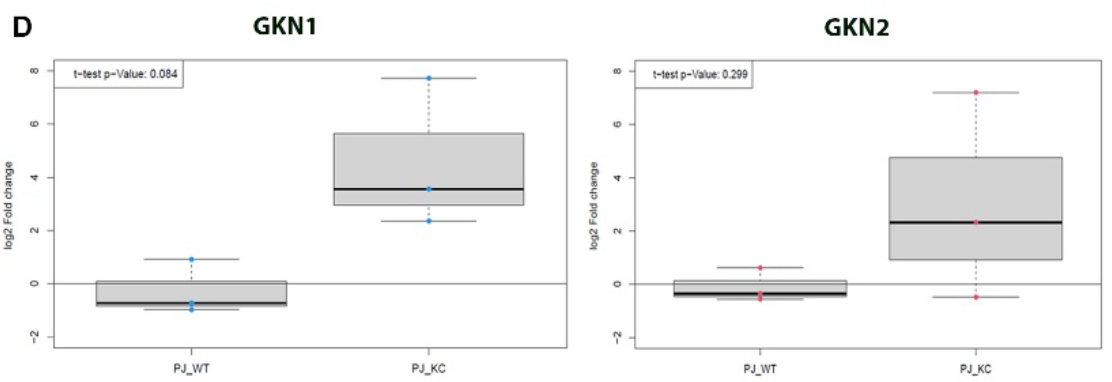
**B**

B

**C**

KC Gkn1KC


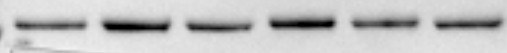


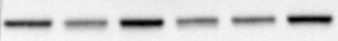

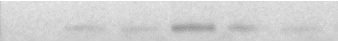

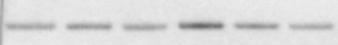


α-Tubulin

# Bcl-2 Mcl-1

Bcl-xL

## Supplemental Figure 3

**Supplemental Figure 3: (A)** Gkn1 and Gkn2 protein expression on western blot in stomach and pancreas of 4 months old wildtype, KC and KPC mice. **(B)** GKN1 and GKN2 protein in mouse pancreatic juice from 6 months old KC and C57BL/6 mice. **(C)** western blot of intrinsic apoptotic pathway components Bcl-2, Mcl-1 and Bcl-xL remain unchanged in KC and Gkn1KC pancreas.


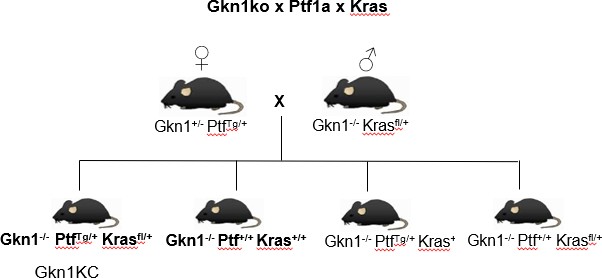

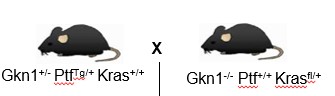


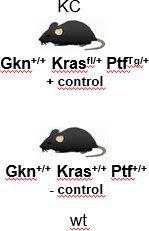


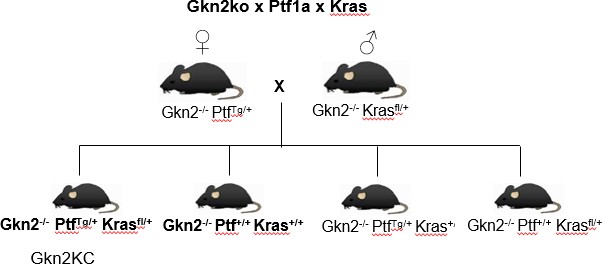

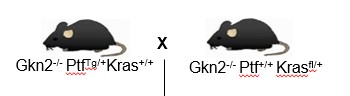


**Supplemental Figure 4**

**Supplemental Figure 4:** Gkn1KC and Gkn2KC breeding scheme. Due to Gkn1-/- females having problems to give birth and resulting increased death of pups, Gkn1+/-;Ptf1aCre/+ females were used for breeding and mated with Gkn1-/-;Kras+/fl males. Thus, we transmit both mutations linked since Gkn1 and Kras are both located on the same arm of chromosome

6. Gkn2KC did not cause breeding difficulties.


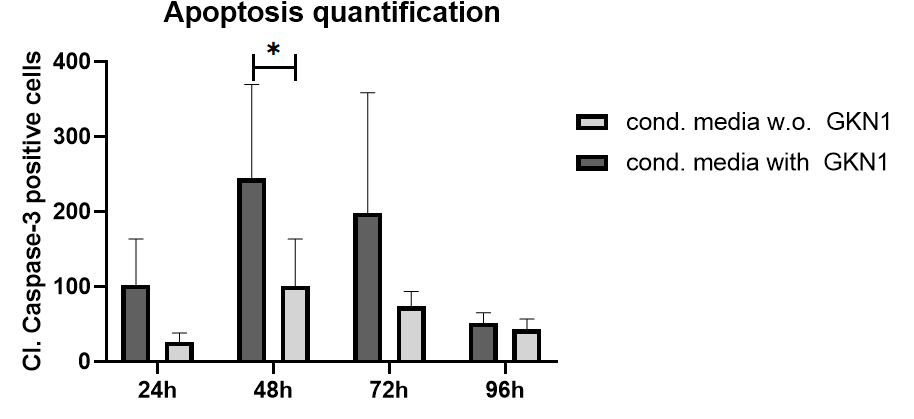


**Supplemental Figure 5**

**Supplemental Figure 5:** Panc02 cells were exposed to either conditioned media without GKN1 or with GKN1 over a period of 24, 48, 72 or 96h. Cells were then stained for Cleaved Caspase-3 and positive stained cells were quantified**.**

## Supplemental Table 1

Patient data for sampling of tissue. The tissue samples were analysed for the expression of GKN. CP: chronic pancreatitis, PDAC: pancreatic ductal adenocarcinoma, (met): metastasis, CA: adeno carcinoma, SCA: serous cystadenoma, SPN: solid pseudopapillary neoplasm, MCN: mucinous cystic neoplasms, AV: Ampulla Vateri, IPMN: intraductal papillary mucinous neoplasms, DA: duodenal adenocarcinoma, DC Ductus Choledochus, PanIN: Pancreatic Intraepithelial Neoplasia, MaSCA: Macrocystic serous adenoma; PT: Peritumoral (possibly more than one sample per patient, adjacent and distant to tumour); T: Tumour. As a result, the highest detectable GKN value is depicted.

**Samples for RNA – GKN expression in pancreatic tumors**

| **Number** | **Gender** | **Site of tissue sampling** | **Histological diagnosis** | **40-Ct GKN1** | **40-Ct GKN2** |
| --- | --- | --- | --- | --- | --- |
| H-2273 | M | PT-T | PDAC | 0 | 0 |
| H-2275 | M | PT-T | PDAC | 6 | 9 |
| H-2277 | M | PT-T | PDAC | 0 | 4 |
| H-2278 | M | PT-T | IPMN | 19 | 19 |
| H-2280 | F | PT-T | PDAC | 4 | 9 |
| H-2282 | F | PT-T | IPMN | 0 | 11 |
| H-2286 | M | PT-T | PDAC | 6 | 6 |
| H-2295 | F | T | PDAC, CP | 0 | 0 |
| H-2299 | M | PT-T | PDAC | 4 | 8 |
| H-2300 | M | PT-T | CA, PanIN | 7 | 10 |
| H-2302 | M | PT-T | PDAC/IPMN | 8 | 10 |
| H-2307 | F | PT-T | PDAC | 11 | 11 |
| H-2308 | F | PT-T | PDAC | 10 | 12 |
| H-2312 | M | PT-T | CA | 0 | 5 |
| H-2314 | M | PT | PDAC | 0 | 3 |
| H-2315 | M | PT | DA, invasive CA | 8 | 10 |
| H-2317 | M | PT -T | PDAC | 10 | 13 |
| H-2320 | F | PT-T | PDAC | 3 | 6 |
| H-2321 | F | PT | CV AV (met) | 14 | 12 |
| H-2323 | M | PT-T | PDAC | 7 | 7 |
| H-2325 | M | PT | PDAC, PanIN | 3 | 3 |
| H-2326 | F | PT -T | PDAC, PanIN | 0 | 3 |
| H-2327 | M | PT -T | PDAC | 3 | 7 |
| H-2328 | M | PT -T | PDAC | 0 | 5 |
| H-2329 | F | PT -T | PDAC, PanIN | 14 | 16 |
| H-2330 | M | PT-T | PDAC, PanIN | 0 | 6 |
| H-2331 | F | PT -T | PDAC, PanIN | 0 | 2 |

1

## Samples for RNA – GKN expression in patients with non-pancreatic malignancies

| **Number** | **Gender** | **Site of**  **tissue sampling** | **Histological diagnosis** | **40-Ct GKN1** | **40-Ct GKN2** |
| --- | --- | --- | --- | --- | --- |
| H-2276 | M | PT | Kidney cell CA (met) | 0 | 0 |
| H-2281 | M | PT | CA AV | 0 | 0 |
| H-2283 | M | PT | CA DC | 0 | 0 |
| H-2285 | M | PT | CP | 0 | 0 |
| H-2287 | M | PT | CA DC | 0 | 0 |
| H-2309 | F | PT-T | malignant melanoma (met) | 0 | 0 |
| H-2310 | F | PT | CP | 0 | 0 |
| H-2316 | M | PT | Cholecystitis, no tumor | 0 | 0 |
| H-2318 | M | PT | CA AV | 0 | 0 |
| H-2322 | F | PT | DA | 0 | 0 |
| H-2324 | M | PT | Peri-amp CA, not pancreatic | 0 | 0 |

**Samples for RNA – GKN expression in patients with adenomas and MCN**

| **Number** | **Gender** | **Site of tissue sampling** | **Histological diagnosis** | **40-Ct GKN1** | **40-Ct GKN2** |
| --- | --- | --- | --- | --- | --- |
| H-2272 | F | PT-T | SCA | 0 | 0 |
| H-2284 | F | PT | MCN | 0 | 0 |
| H-2289 | F | PT | MCN | 0 | 0 |
| H-2296 | M | PT -T | SCA | 0 | 3 |
| H-2304 | F | T | SPN | 0 | 0 |
| H-2319 | F | PT | MaSCA | 0 | 0 |

**Samples for RNA– controls from healthy patients with healthy pancreas**

| **Number** | **Gender** | **Site of tissue sampling** | **Diagnosis** | **40-Ct GKN1** | **40-Ct GKN2** |
| --- | --- | --- | --- | --- | --- |
| 1 | F | H | multiorgan transplantation | 0 | 0 |
| 2 | M | H | multiorgan transplantation | 0 | 0 |
| 3 | M | H | multiorgan transplantation | 0 | 0 |
| 4 | M | H | multiorgan transplantation | 0 | 0 |
| 5 | F | H | multiorgan transplantation | 0 | 0 |
| 6 | M | H | multiorgan transplantation | 0 | 0 |
| 7 | F | H | Polytrauma patient | 0 | 0 |
| 8 | M | H | Duodenal resection with healthy pancreas attached | 0 | 0 |

2

## Supplemental Table 2

**PanIN gene expression analysis at 3 months:**

| **Gene category** | **Gene** |  | **Gene category** | **Gene** |
| --- | --- | --- | --- | --- |
| Genes associated with Gastrokines and PanIN relevant | CCKBR |  | Tissue remodelling | Mmp7 |
|  | Gkn1 |  | Apoptosis | Bcl-2 |
|  | Gkn2 |  | Infiltrating cells | B220 |
|  | Tff1 |  |  | CD3 |
|  | Cld18 |  |  | Emr1 |
|  | Cyclin D1 |  | Inflammatory Cytokines | IL2 |
|  | Sox9 |  |  | IL4 |
|  | p16 |  |  | IL10 |
|  | p21 |  |  | Il6 |
|  | Mist1 |  |  | Il1b |
|  | aSMA |  |  | Tnfa |
|  | Muc5ac |  | EMT marker- epithelial | Muc1 |
|  | Muc6 |  |  | E-cad |
|  | Sparc |  |  | Col4a1 |
|  | Hspa1a |  | EMT marker- mesenchymal | Vim |

3

## Supplemental methods:

## Mass Spectrometry - Protein Identification and Quantification using Progenesis QI for Proteomics

Sample preparation: Samples were diluted 1:10 in SDS buffer (4% SDS, 100 mM Tris / HCL pH 8.2, 0.1M DTT – dithiothreitol) boiled at 95°C for 5 minutes and processed with High Intensity Focused Ultrasound (HIFU) for 10 mins setting the ultrasonic amplitude to 65%. Protein concentration was then estimated using the Qubit® Protein Assay Kit (Life Technologies, Zurich, Switzerland). For each sample, 10 µg of proteins were taken and used for on-filter digestion using an adaptation of the filter-aided sample preparation (FASP) protocol [1]. Briefly, proteins were diluted in 200 µl of UT buffer (Urea 8M in 100 mM Tris/HCL pH 8.2), loaded on Ultracel 30000 MWCO centrifugal unit (Amicon Ultra, Merck, Darmstadt, Germany) and centrifuged at 14000g. SDS buffer was exchanged by one centrifugation round of 200 µl UT buffer. Alkylation of reduced proteins was carried by 5 min incubation with 100 µl iodoacetamide 0.05M in UT buffer, followed by three 100 µl washing steps with UT and three 100 µl washing steps with NaCl 0.5M. Finally, proteins were on-filter digested using 120 µl of 0.05 Triethylammonium bicarbonate buffer (pH 8) containing trypsin (Promega, Madison, WI, USA) in ratio 1:50 (w/w). Digestion was performed overnight in a wet chamber at room temperature. After elution, the solution containing peptides was acidified to a final 0.1% TFA, 3% acetonitrile concentration. Peptides were desalted using Waters SPE C18 columns (Waters AG, Switzerland), dried and re-solubilized in 15 µl of 3% acetonitrile, 0.1% formic acid for MS analysis.

The raw files from the mass spectrometer were loaded into ProgenesisQI for Proteomics (v.4.0). The aligning reference was chosen as such where the most features could visually been identified. From each Progenesis PeptideIon (default sensitivity in peak picking) a maximum of the top five tandem mass spectra were exported using charge deconvolution and deisotoping option and a maximum number of 200 peaks per MS/MS. The Mascot generic file (.mgf) was searched with Mascot Server v.2.5.1 (www.matrixscience.com) using the parameters 10ppm for precursor ion mass tolerance, 0.6 Da for fragment ion tolerance. Semi- trypsin option was used as the protein-cleaving enzyme, one missed cleavage was allowed. Carbamidomethylation of cysteine was specified as a fixed modification, and oxidation of methionine, pyroglutamate formation from glutamine, deamidation from glutamine and asparagine and N-terminal acetylation of proteins were selected as variable modifications.Searched was a forward and reversed mouse and bovine database (mouse downloaded on 2014/07/15 from uniprot,) concatenated to 260 known mass spectrometry contaminants in order to evaluate the false discovery rate using the target-decoy strategy. The mascot result was loaded into Scaffold v4.1.1 using 5% peptideFDR and 10% proteinFDR thresholds and protein cluster analysis. The spectrum report was exported and loaded into ProgenesisQI for proteomics.

In the experimental design we used a between group analysis where the relevant experimental design was generated. A) Control group (n=3) and KC (n=3). Normalization was kept with default settings.

For quantification we assed all proteins identified with at least 2 features. Proteins were grouped with Progenesis. For protein quantification, the average of the normalised abundance from the most intense three peptide ions of each protein group were calculated individually for each sample [2]. This generates the normalised quantitative protein abundance. For statistical testing the parametric test (analysis of variance) on the transformed (hyperbolic arcsine transformation) normalised protein abundance was applied. The mass spectrometry proteomics data were handled using the local laboratory information management system (LIMS).

Focusing on the gastrokine proteins only the normalized protein abundances as reported by Progenesis were used and the log2 fold changes to the mean expression of the WT samples were reported.

## Microdissection and aRNA-longSAGE library production

Surgical pancreatic resection specimens were immediately placed on ice, and tissue from the carcinoma, peritumoral parenchyma and resection margins was removed, snap frozen and stored at -80°C. For the identification of normal ducts and PanINs, 5 µm frozen sections prepared from tissue blocks of peritumoral pancreatic parenchyma, in particular from the resection margins, were briefly placed in RNase free ethanol (Merck, Darmstadt, Germany), stained with H&E and diagnosed by a pathologist (J.L., G.K.). PanINs were classified according to published criteria (30). PanIN lesions were manually microdissected under a microscope (BH2, Olympus, Wetzlar, Germany) using a sterile injection needle (size 0.65x25 mm, Fa. Braun, Melsungen, Germany). Medium-sized interlobular ducts were selected by preference in order to avoid contamination by acinar tissue. Microdissected cells in 50 µl extraction buffer (Arcturus, Moerfelden-Walldorf, Germany) were kept on ice. From normal ductal epithelium or PanIN-1B, PanIN-2 and PanIN-3 lesions that were present in serial sections 2000-3500 cells each were generally collected and pooled. Each cell pool contained cells from four to eight different cases.

## aRNA-longSAGE

Libraries were generated as described previously [3]. Except for some minor modifications second strand synthesis and all the other steps of longSAGE library generation were done according to the MicroSAGE protocol version 1.0e (<http://www.sagenet.org/protocol>

/index.htm) adapted for the longSAGE protocol published by Saha et al. 3. The aRNA- longSAGE libraries consisted of more than 10,000 clones with an insert length of > 600 basepairs. Sequencing of the libraries was performed by MWG (MWG Biotech, Ebersberg, Germany). LongSAGE tags were extracted from the sequence files with SAGE-PHRED 2003 software (which can be obtained from je@bio.aau.dk): the minimum quality of each base within a tag sequence was set to PHRED20 [4] and the maximum ditag length was set to 36 (not including flanking CATGs). For tag annotation, tag sequences were extracted from different data sources to create local tag databases. The local databases contained the 3´- most tags of all sequences from RefSeq release 6, the mammalian gene collection (designated “MGC”) and the TIGR EST assembly database.

**Western blot**

Immunoblotting was performed by homogenizing tissue samples in RIPA buffer containing protease inhibitor cocktail (Roche Diagnostics, Mannheim, Germany) and 1mM of PMSF. Protein concentrations were determined using a DC Protein Assay Reagents Package #5000116 (BioRad, Hercules, CA, USA). Protein aliquots were separated by SDS–PAGE electrophoresis and blotting, using a V3 Western Workflow system (BioRad, Hercules, CA, USA), according to the manufacturer's protocols and run along a PageRuler Plus, prestained protein ladder (26619,Thermo Scientific). PVDF membranes were blocked in TBST containing 5% BSA and incubated with primary antibodies overnight at 4°C. Proteins of pancreas homogenates were measured by densitometry and shown by expression relative to GAPDH or α-Tubulin as the reference protein. Serum samples were prepared as follows: Mouse blood from 9-month-old animals was collected and left for 1h at RT for coagulation to occur. Blood was centrifuged for 5 min at 7500g and serum was aspirated without touching the blood clot. For western blot analysis, 80mg of serum proteins were heated to 95°C for 5 min and run on gel. Serum sample results were normalized to the total protein content per lane.

**Collection of Pancreatic Juice**

Mice were anesthetized with isoflurane. A right upper quadrant laparotomy was performed, and the duodenum was identified. The bile duct was ligated just above the pancreatic duct in order to avoid bile collection through the common pancreato-biliary duct. Polyethylene tubing (0.28 mm inside diameter × 0.61 mm outside diameter; PE-10) was inserted into the common bile duct at the ampulla of Vateri, and pancreatic juice was collected for 60-minute periods into tubes kept on ice.

**Microarray analysis**

Three animals per strain and time point were tested. Total RNA samples quality was determined with a NanoDrop ND 1000 (NanoDrop Technologies, Delaware, USA) and a Bioanalyzer 2100 (Agilent, Santa Clara, California, United States). Only those samples with a 260 nm/280 nm ratio between 1.8–2.1 and a 28S/18S ratio within 1.5–2 were further processed. RNA samples (100ng) were reverse transcribed into cDNA using the Ambion WT Expression Kit (PN442509D) and further processed accordingly GeneChip® WT Terminal Labeling and Hybridization User Manual for use with the Ambion® WT Expression Kit (P/N702808). The array used were GeneChip™ Mouse Gene 2.1 ST Array Strip (Thermofisher Scientific 902120), hybridized in a GeneAtlas Hybridization Station for 20 h at 48°C. Arrays were then washed using Affymetrix GeneAtlas Fluidics Station. An Affymetrix GeneAtlas Imaging Station was used to measure the fluorescent intensity emitted by the labeled target.

**References**

1 Wiśniewski JR, Zougman A, Nagaraj N, Mann M. Universal sample preparation method for proteome analysis. *Nature Methods* 2009; 6: 359-362.

2 Grossmann J, Roschitzki B, Panse C, Fortes C, Barkow-Oesterreicher S, Rutishauser D *et al*. Implementation and evaluation of relative and absolute quantification in shotgun proteomics with label-free methods. *J Proteomics* 2010; 73: 1740-1746.

3 Saha S, Sparks AB, Rago C, Akmaev V, Wang CJ, Vogelstein B *et al*. Using the transcriptome to annotate the genome. *Nature biotechnology* 2002; 20: 508-512.

4 Ewing B, Green P. Base-calling of automated sequencer traces using phred. II. Error probabilities. *Genome research* 1998; 8: 186-194.
